# Endangered Pinto/Northern Abalone ( *Haliotis kamtschatkana* ) are Panmictic Across Their 3700 km Range Along the Pacific Coast of North America

**DOI:** 10.1111/eva.70040

**Published:** 2024-12-03

**Authors:** J. L. Dimond, J. V. Bouma, F. Lafarga‐De la Cruz, K. J. Supernault, T. White, D. A. Witting

**Affiliations:** ^1^ Shannon Point Marine Center Western Washington University Anacortes Washington USA; ^2^ Puget Sound Restoration Fund Bainbridge Island Washington USA; ^3^ Centro de Investigaciones Científicas y de Educación Superior de Ensenada Ensenada Baja California Mexico; ^4^ Fisheries and Oceans Canada, Pacific Biological Station Nanaimo British Columbia Canada; ^5^ University of California Santa Cruz Santa Cruz California USA; ^6^ NOAA National Marine Fisheries Service, Office of Habitat Conservation, Restoration Center Long Beach California USA

**Keywords:** genetic connectivity, *Haliotis kamtschatkana*, marine conservation, population genomics

## Abstract

Connectivity is integral to the dynamics of metapopulations through dispersal and gene flow, and understanding these processes is essential for guiding conservation efforts. Abalone, broadcast‐spawning marine snails associated with shallow rocky habitats, have experienced widespread declines, and all seven North American species are threatened. We investigated the connectivity and population genomics of pinto/northern abalone ( *Haliotis kamtschatkana* ), the widest‐ranging of abalone species. We employed reduced representation sequencing (RADseq) to generate single nucleotide polymorphism (SNP) data, assessing population connectivity and potential adaptive variation at 12 locations across the full range from Alaska to Mexico. Despite depleted populations, our analysis of over 6000 SNPs across nearly 300 individuals revealed that pinto abalone maintains a high genetic diversity with no evidence of a genetic bottleneck. Neutral population structure and isolation by distance were extremely weak, indicating panmixia across the species' range (global *F*_ST_ = 0.0021). Phylogenetic analysis, principal components analysis, and unsupervised clustering methods all supported a single genetic population. However, slight population differentiation was noted in the Salish Sea and Inside Passage regions, with evidence for higher barriers to dispersal relative to outer coastal areas. This north‐central region may also represent the species' ancestral range based on relatively low population‐specific *F*_ST_ values; the northern and southern extremes of the range likely represent range expansions. Outlier analysis did not identify consensus loci implicated in adaptive variation, suggesting limited adaptive differentiation. Our study sheds light on the evolutionary history and contemporary gene flow of this threatened species, providing key insights for conservation strategies, particularly in sourcing broodstock for ongoing restoration efforts.

## 1 Introduction

Connectivity is a cornerstone concept in ecology and evolution, as it relates to the dynamics of metapopulations through migration and dispersal of individuals. It is particularly crucial in facilitating gene flow, shaping genetic diversity within and among populations. The exchange of genes has far‐reaching implications, affecting a species' ability to adapt to changing environments and the broader trajectory of its evolution (reviewed by Cramer et al. 2023).

The current biodiversity crisis underscores the importance of studying and understanding species connectivity. Information on species connectivity can guide conservation efforts, such as creating protected areas that connect fragmented habitats and allow species' natural movement and dispersal (Palumbi 2003). It also aids in developing conservation strategies that are tailored to the genetic health and adaptive potential of populations, ensuring that they can continue to breed effectively and withstand environmental changes (Hoffmann et al. 2015). Integrating our understanding of connectivity and population structure into conservation planning is therefore essential for the survival of individual species and the maintenance of biodiversity and ecosystem health.

Many marine organisms have a life history that includes a dispersive planktonic stage that serves as their primary means of population connectivity and gene flow. While the planktonic stage can facilitate widespread dispersal, several factors can influence realized connectivity, including larval duration, larval behavior, ocean currents, and physical barriers (Cowen and Sponaugle 2009). Moreover, selective forces can promote local adaptation and genomic divergence even in the face of strong connectivity (Tigano and Friesen 2016). Empirical studies have revealed a range of scenarios among marine organisms; some show extensive connectivity across large geographic areas, while others display significant population structuring, suggesting that the connectivity of marine species is a complex interplay of biological and physical factors that can vary widely among species (Cowen and Sponaugle 2009). Understanding these dynamics is crucial for marine conservation, particularly in designing marine protected areas, managing fisheries, and restoring depleted populations, where knowledge of population connectivity can influence the success of conservation and management efforts.

Abalone, molluscs in the Family Haliotidae, are large, herbivorous, broadcast‐spawning marine snails that occur worldwide on shallow temperate and tropical rocky reefs. Overharvest, disease, and other factors have taken a toll on many species of abalone, and all seven extant abalone species along the Pacific Coast of North America are now considered endangered (IUCN Red List of Threatened Species 2022). Pinto, or northern, abalone ( *Haliotis kamtschatkana* Jonas, 1845) are the widest‐ranging North American species, occurring from Mexico to Alaska. Decades of overharvest pushed pinto abalone populations to the brink of collapse throughout this range, and by the 1990s, fishery closures were implemented in Alaska, British Columbia, Washington, and California (Neuman et al. 2018; Peters and Rogers‐Bennett 2021). Since then, most populations have slightly recovered, with limited natural recruitment observed only in Alaska and British Columbia (Peters and Rogers‐Bennett 2021). In Washington, where commercial harvest was never permitted, pinto abalone continued to decline even after the closure of a significant recreational fishery and are now at only ~3% of their pre‐closure numbers, when the population was already considerably depleted (Carson and Ulrich 2019; Rothaus, Vadopalas, and Friedman 2008). Meanwhile, size frequency distributions of abalone over time indicate that abalone are aging out of the population with no new recruitment (Bouma et al. 2012; Carson and Ulrich 2019). These data suggest pinto abalone in Washington are experiencing reproductive failure due to an Allee effect, whereby the density of breeding adults is too low for successful reproduction (Bouma et al. 2012; Carson and Ulrich 2019; Rothaus, Vadopalas, and Friedman 2008).

Despite depleted populations throughout their range, pinto abalone appear to have maintained a high degree of genetic variation and no evidence of a genetic bottleneck (Dimond, Bouma et al. 2022; Withler et al. 2003). Withler et al. (2003) used microsatellites to evaluate genetic variation and population structure among *H. kamtschatkana* throughout British Columbia and Alaska and found high gene diversity and effective population size with only weak population structure. More recently, analyzing thousands of single nucleotide polymorphisms (SNPs), (Dimond, Gathright et al. 2022) corroborated these results based on comparisons of wild abalone from one Washington and one Alaska locale, finding high allelic richness and effective population size and very low genomic divergence between locations separated by ~1000 km. Though focused on the northern extent of the species' range, these findings suggest that historically, *H. kamtschatkana* did not function as isolated population units and that there is no evidence of disrupted gene flow due to recent declines in abundance.

A full appraisal of range‐wide population structure is essential to provide a more complete picture of *H. kamtschatkana* population genomics and support conservation efforts. In Washington, *H. kamtschatkana* restoration efforts began in the mid‐2000s, and thousands of captive‐bred juvenile abalone have been released at multiple restoration sites. Although current efforts utilize locally collected wild broodstock abalone from Washington waters (Carson et al. 2019; Dimond, Gathright et al. 2022), local population declines are severe enough that locating individuals is increasingly difficult (Dimond, Bouma et al. 2022). To enable continued restoration efforts, broodstock from beyond Washington may need to be sourced. Identifying suitable non‐local broodstock requires an improved understanding of range‐wide pinto abalone population genetics. Knowledge of population genetic structure would permit critical decision making regarding the relative risks of inbreeding due to limited local broodstock versus outbreeding resulting from the introduction of outsourced broodstock to the breeding program. Here, we expand our understanding of pinto abalone connectivity and population structure by analyzing the full range of the species from Alaska to Mexico. We used reduced representation sequencing to generate SNP marker data with which to evaluate both population connectivity and potential adaptive variation.

## 2 Methods

### 2.1 Specimen Collection

**FIGURE 1:**
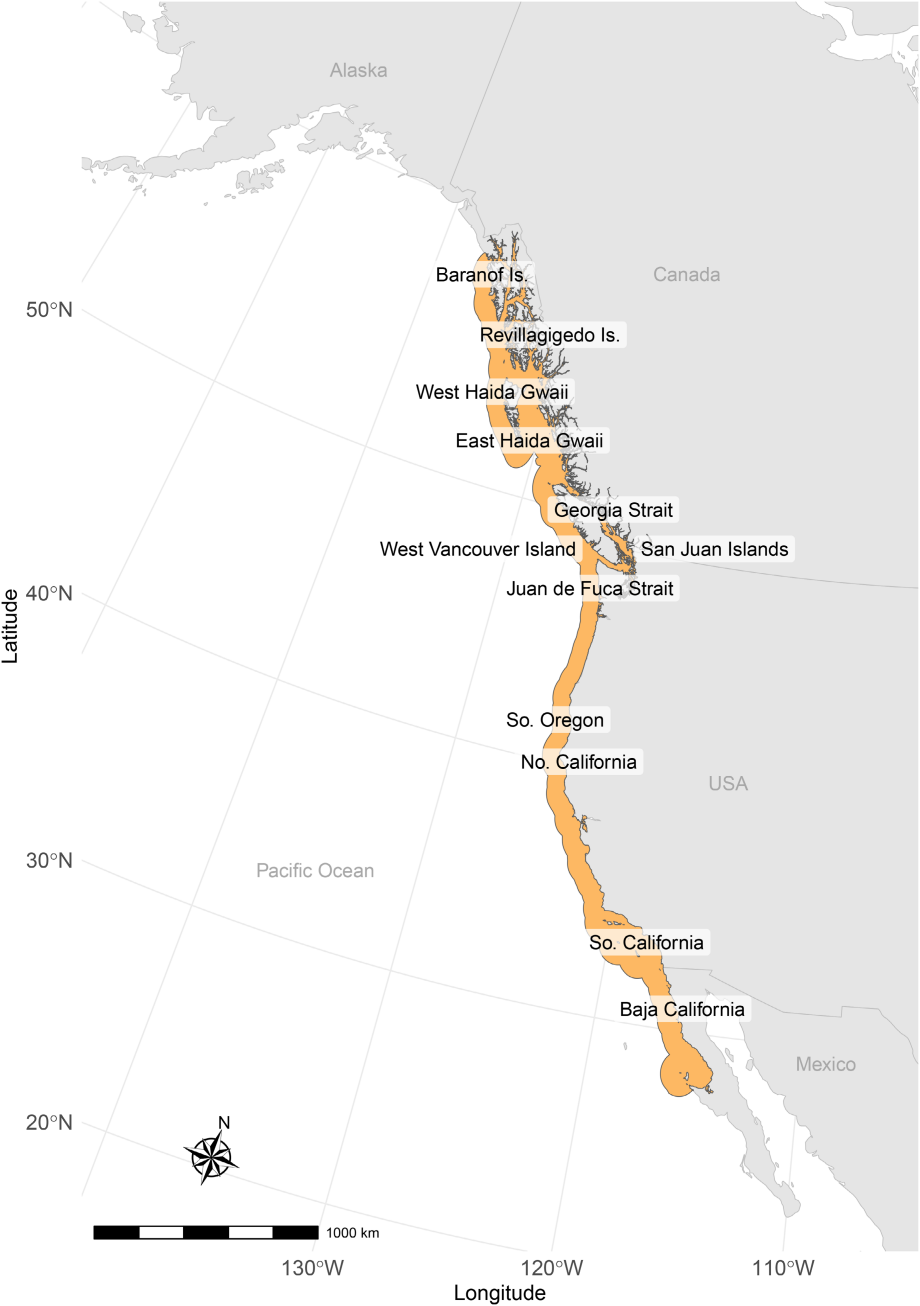
Geographic range of *H. kamtschatkana* along the Pacific coast of North America shown in orange, with labels indicating the locations of samples taken for genomic sequencing.

Pinto abalone were sampled from 12 locations along their 3700 km geographic range between 2013 and 2021 (Figure 1). In most cases, pinto abalone tissue samples were obtained non‐lethally by divers using scissors to excise abalone epipodial tentacles in situ. In all cases, tissue was preserved in 90%–100% ethanol and kept refrigerated prior to DNA extraction. The geographic locations of collection sites are intentionally vague due to the species' protected status. For mapping, the geographic range of *H. kamtschatkana* was downloaded as a shapefile from the IUCN Red List website (IUCN 2021).

### 2.2 DNA Extraction and Sequencing

The Qiagen DNeasy Blood and Tissue 96‐well kit was used for DNA extraction, following overnight lysis in Proteinase K. DNA quality was spot‐checked on a subset of samples with 1% agarose gel electrophoresis. DNA concentration across all samples was tested with the QuantiFluor ONE dsDNA System (Promega) and samples were normalized to approximately 20 ng/μL before shipping to Floragenex Inc. (Beaverton, Oregon) for DNA library preparation and sequencing. Libraries were prepared using the restriction site‐associated DNA sequencing technique (RADseq; (Baird et al. 2008)) with restriction enzyme *SbfI* and sequenced on two NovaSeq 6000 SP flow cells with 2 × 150 bp output.

### 2.3 Bioinformatic Data Processing

The *Stacks* v.2.6 *process_radtags* program (Rochette, Rivera‐Colón, and Catchen 2019) was used with default settings to filter and demultiplex raw reads. The resulting demultiplexed reads were mapped to the red abalone ( *H. rufescens* ) reference genome (Masonbrink et al. 2019) using *BWA mem* in bwa v.0.7.17 (Li and Durbin 2009) with default settings. Red abalone is closely related to pinto abalone, and due to the shallow divergence between the two species, the use of this genome for read mapping is appropriate (Dimond, Gathright et al. 2022; Masonbrink et al. 2019). High‐quality mapped reads with mapQ 20+ and properly‐paired reads were sorted, converted to BAM format using *samtools* (Li et al. 2009) v.1.19, and assembled with the *Stacks gstacks* program. Final SNP calls were output by the *Stacks populations* program using the PCR duplicate removal option, a minimum minor allele count of 3 to process a locus and the requirement that a locus occurred in a minimum of 50% of individuals in a population in order to process it. The resulting Stacks VCF file underwent further SNP filtering using the R package *vcfR* v1.13.0 (Knaus and Grünwald 2017). Variants falling outside 95% confidence intervals in sequencing depth were removed, along with those possessing Phred quality scores below 20 and exhibiting over 40% missing data. Samples with greater than 80% missing data were also excluded. Lastly, the R package *SNPRelate* v1.20.1 (Zheng et al. 2012) was used to perform LD‐based SNP pruning, with an LD threshold of *r* ^2^ = 0.2, in order to exclude variants based on linkage disequilibrium. Finally, to assess assembly quality and genotyping error in the final SNP dataset, SNP mismatches were computed between 62 samples that were sequenced in duplicate, serving as technical replicates. Technical replicates were excluded from the dataset for subsequent analyses.

### 2.4 Data Analysis

We computed population statistics, including gene diversity (*H*_S_), observed heterozygosity (*H*_O_), inbreeding coefficients (*F*_IS_), allelic richness (AR), global *F*_ST_, and population‐specific *F*_ST_ with the R packages *dartR* v.2.9.7 and *hierfstat* (Goudet 2005; Mijangos et al. 2022). Effective population size (*N*_e_) was calculated using the LD method in *NeEstimator* v2 (Do et al. 2014); we report estimates at an allele frequency of 0.01. A phylogenetic tree was rendered with the R package *ape* v.5.7‐1 using the UPGMA method (Paradis and Schliep 2019). Two independent methods were used to assess population differentiation without priors: (1) sparse nonnegative matrix factorization (*snmf*) implemented in the R package *LEA* v3.14.0 (Frichot and François 2015), and (2) the *k*‐means clustering procedure *find.clusters* implemented in the R package *adegent* 2.1.10 (Jombart 2008). Both procedures were tested with a range of *k* = 1–10, with cross‐entropy values and the Bayesian information criterion used, respectively, to evaluate optimal *k*. Principal components analysis on the SNP dataset was performed with *adegent* 2.1.10 (Jombart 2008). Finally, to assess relatedness, a genomic relatedness matrix was computed with the R package *StAMPP* 1.6.3 (Pembleton and Cogan 2013) based on the method of Yang et al. (2010).

We used two different methods to identify outlier SNP markers: (1) the R package *pcadapt* v.4.3.3 (Luu, Bazin, and Blum 2017) with a principal components‐based method and (2) the R package *OutFLANK* v0.2 (Whitlock and Lotterhos 2015) which identifies outliers based on the *F*_ST_ distribution. Loci identified as outliers by both methods were considered as outliers for further analysis.

Pairwise *F*_ST_ was calculated with 100 bootstrap replicates and assessed for significance based on 95% confidence intervals using the method of Reich et al. (2009) with an R script by (Junker et al. 2020). This *F*_ST_ method is particularly robust for population sample sizes as low as *N* = 2 (Willing, Dreyer, and van Oosterhout 2012). We tested for isolation by distance (IBD) by creating a pairwise matrix of lineal water distances between sites and compared the distance matrix and the *F*_ST_ matrix with a Mantel test using the R package *ade4* v.1.7‐20 (Dray and Dufour 2007).

To uncover spatial gene flow patterns, we used the Python package *feems* (Marcus et al. 2021) to estimate effective migration surfaces. Based on a stepping‐stone model, this approach identifies migration rates between populations that differ from those anticipated under pure IBD, thus pinpointing barriers or corridors of gene flow. A discrete global grid for the study area was made by clipping a grid created in the R package *ddgridR* (Barnes 2018) to a simplified polygon of the *H. kamtschatkana* range (IUCN 2021).

## 3 Results

The final assembly contained a total of 285 samples and 6791 SNPs with 33.5% missing data. Sample sizes in the final assembly varied between a low two in Southern Oregon to a high of *N* = 69 in the San Juan Islands (Table 1). Generally, sample sizes were higher in northern regions than in southern regions. Technical replicates (samples sequenced as independent, duplicate libraries) allowed us to estimate a genotyping error of 0.6%, indicating that the final assembly was of very high quality, with 99.4% overall multilocus genotyping accuracy. Each site displayed comparable levels of observed (*H*_O_; 0.054–0.059) and expected heterozygosity (*H*_S_; 0.045–0.056), with no indications of heterozygote deficiencies (Table 1). However, heterozygosity was lowest at the southern and extreme northern limits of the range. Population‐specific *F*_ST_, which reflects ancestral populations and geographic range expansions (Kitada, Nakamichi, and Kishino 2021), ranged from −0.087 at Revillagigedo Island to 0.110 in Northern California (Table 1). Generally, lower *F*_ST_ values occurred among populations from the north‐central portion of the range, whereas the extremities of the range showed higher values. Global *F*_ST_ for the entire dataset was very low at 0.0021. Allelic richness (AR) was higher among north‐central populations while lower at the range extremes. Effective population size (*N*_e_) is shown for minor allele frequencies of 0.01 and was estimated to be infinite for all populations except Revillagigedo Is. (399) and the San Juan Islands (1312).

**TABLE 1:**
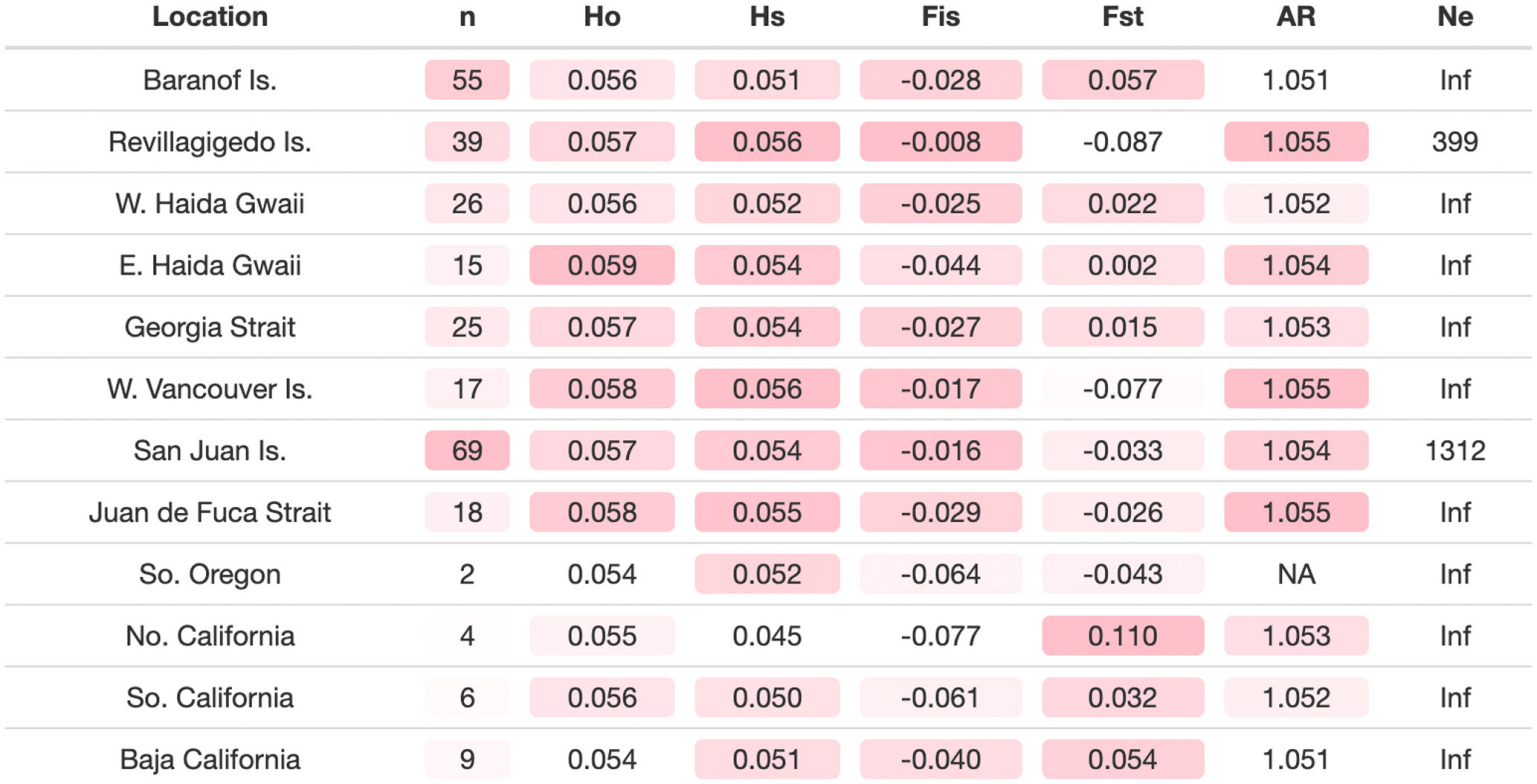
Summary statistics for *H. kamtschatkana* populations sampled in this study.

*Note:* For each column, pink shading is used to highlight high (darker shading) and low (low or no shading) values.

Abbreviations: AR, allelic richnes; *F*_IS_, inbreeding coefficient; *F*_ST_, population‐specific fixation index; *H*_O_ , observed hterozygosity; *H*_S_, gene diversity/expected heterozygosity; *N*_e_, effective population size.

**FIGURE 2:**
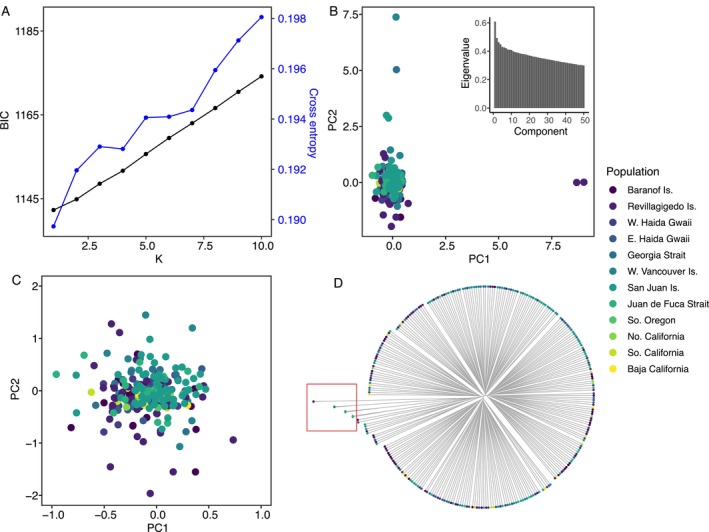
(A) Results of *snmf* and *find. clusters* analyses, showing cross entropy and Bayesian information criterion (BIC) in relation to *k* ranging from 1 to 10. (B) Principal components analysis of all samples with inset scree plot showing component loadings. Samples are colored according to the population color scheme in right‐hand legend. (C) Zoomed‐in view of (B) excluding outliers determined to be close relatives. (D) Dendrogram of the genomic relatedness distance matrix, showing highlighted outliers likely to be close relatives. Most of these individuals also appear as outliers in (B).

Multiple analyses failed to detect population structure in *H. kamtschatkana*. The *snmf* and *find. clusters* procedures indicated that *k =* 1 had both the lowest cross entropy value and the lowest Bayesian information criterion, respectively (Figure 2A). Similarly, PCA suggested no population structure, with most samples forming a single cluster and a prominent “elbow” in component eigenvalues beyond *k =* 1 (Figure 2B). Outlier samples in the PCA (excluded in Figure 2C) were determined to be close relatives based on relatedness analysis (Figure 2D). This analysis identified pairs of putative relatives at Revillagigedo Is., Juan de Fuca Strait, the San Juan Islands, and intriguingly, between Georgia Strait and West Vancouver Island, a water distance of 370 km. To determine if close relatives were strongly biasing the PCA and obscuring potential population structure, we ran the PCA excluding one individual from each pair and found no effect on lack of structure. Based on these results and the recommendations of Waples and Anderson (2017) we opted not to exclude relatives from any of the analyses.

**FIGURE 3:**
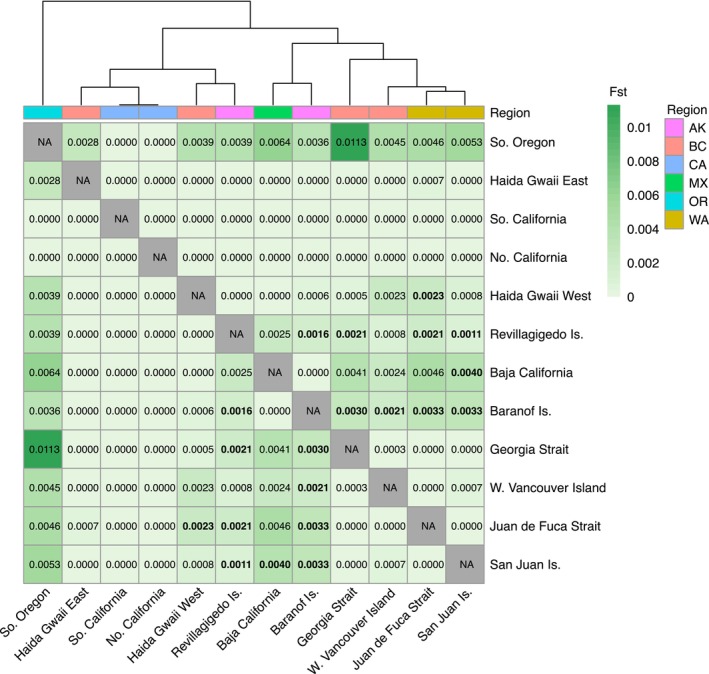
Hierarchically clustered heatmap of pairwise *F*_ST_ values by location. Significant values are shown in bold italics. Region abbreviations: AK, Alaska; BC, British Columbia; CA, California; MX, Mexico; OR, Oregon; WA, Washington.

**FIGURE 4:**
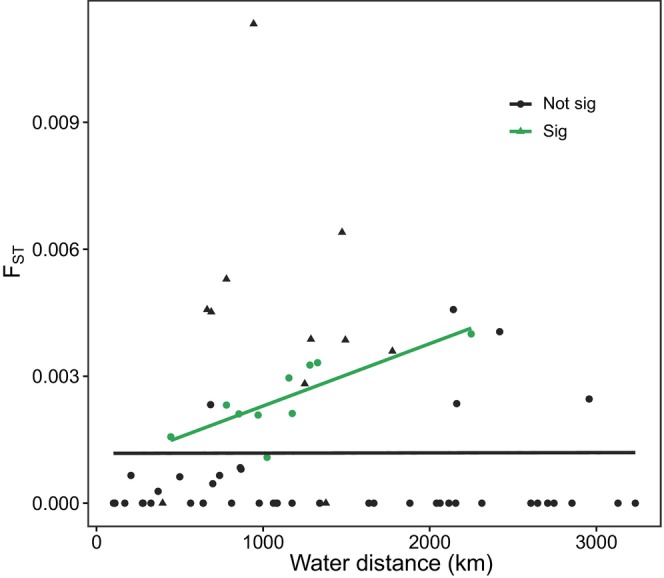
Analysis of isolation by distance (IBD), with pairwise lineal water distance between sampling locations on the *x*‐axis and pairwise *F*_ST_ values on the *y*‐axis. The all‐inclusive analysis is shown in black (Mantel test, *p* = 0.41), while the analysis including only significant pairwise *F*_ST_ values is shown in green (Pearson test, *p* = 0.007). Outlier values from pairwise comparisons including Southern Oregon are shown as black triangles.

Pairwise *F*_ST_ analysis also showed limited evidence of population structure in *H. kamtschatkana* , with most pairwise *F*_ST_ values below 0.005 and only a few of them significant (Figure 3). Pairwise comparisons with Southern Oregon exhibited the highest *F*_ST_ values. Still, these were non‐significant and likely an artifact of the particularly low sample size there. Some regions clustered together as expected under IBD, mostly notably the Salish Sea region encompassing Georgia Strait, the San Juan Is., Juan de Fuca Strait, and W. Vancouver Is. These areas showed no significant differences between each other but were among the few areas that exhibited significant *F*_ST_ values (*F*_ST_ = 0.0011–0.0040) when compared with other regions including Baranof Is., Revillagigedo Is., Baja California, and West Haida Gwaii. Analysis of IBD using all locations exhibited a slope near zero and was non‐significant (Mantel test, *p* = 0.41), but when only significant pairwise *F*_ST_ values were included, the slope was positive and significant (Pearson test, *p* = 0.007; Figure 4).

Outlier detection with *OutFLANK* and *pcadapt* failed to identify consensus loci that could represent putative adaptive variants. Thirty‐two outlier loci were identified by *pcadapt*, but no outliers were detected by *OutFLANK*.

**FIGURE 5:**
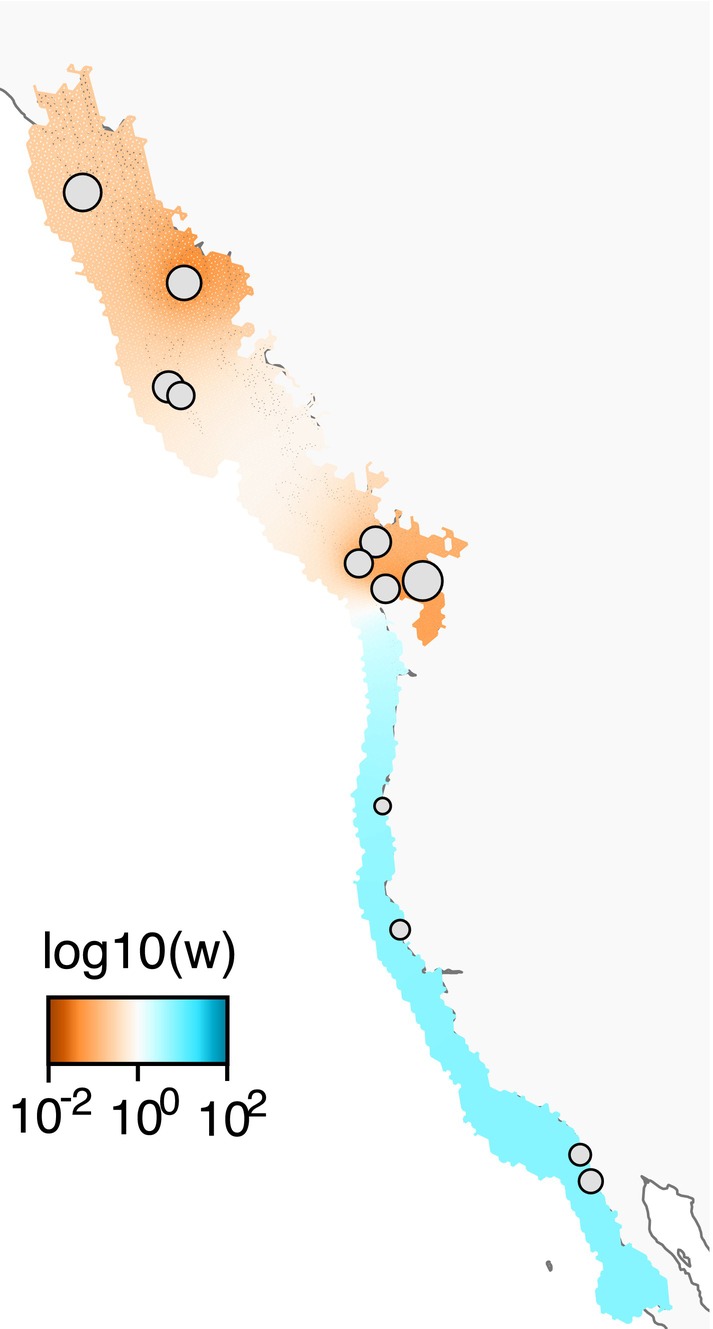
Results of *feems* (fast estimation of effective migration surfaces) analysis along the range of *H. kamtschatkana*, showing estimated surfaces for log of effective migrants per generation. Gray circles represent sampling locations, with the size of the circle relative to the sample size.

*Feems* analysis showed a general pattern of relatively low migration rates north of Washington and higher rates to the south (Figure 5). The analysis pinpointed two areas as relative dispersal barriers: the Salish Sea and Revillagigedo Is.

## 4 Discussion

Our range‐wide study of *H. kamtschatkana* found extremely weak neutral population structure and failed to detect adaptive variation, suggesting that the species is panmictic across its 3700 km range. Instances of range‐wide panmixia in the sea are relatively rare (Lourenço et al. 2017), although there may be reporting bias toward studies that challenge the conventional paradigm of high gene flow (Bierne, Bonhomme, and Arnaud‐Haond 2016). While our study is not the first to find a lack of population structure in a broadcast‐spawning marine invertebrate across the Pacific Coast of North America (e.g., Addison et al. 2008; Kelly and Palumbi 2010), it is the only study to date that we are aware of to document such a lack of population structure using genome‐wide SNP markers. Our results corroborate and extend earlier work by Withler et al. (2003) and Dimond, Bouma et al. (2022), which found little population differentiation across Washington, British Columbia, and Alaska. Furthermore, we found no evidence of restricted gene flow suggestive of incipient speciation; the existence of a subspecies, *H. kamtschatkana assimilis* , was long suspected in the southern part of the range but was recently refuted by examination of both morphological and molecular evidence (Owen and Raffety 2017; Supernault et al. 2010). Our data further support these conclusions.

Varying degrees of population genetic structure have been documented among other species of abalone. For example, there is clear population differentiation among *H. discus* in Korea and Japan, suggesting that geographic and oceanographic barriers can restrict connectivity in abalone (Nam et al. 2021). On the other hand, some studies have identified adaptive genetic structure in abalone without corresponding neutral genetic patterns, implying that selective pressures can drive genetic differentiation despite high gene flow in some circumstances (De Wit and Palumbi 2013; Miller et al. 2019; Sandoval‐Castillo et al. 2018). There is also evidence that both neutral and adaptive genetic structure in abalone can arise over just a few hundred kilometers (Mares‐Mayagoitia et al. 2021). Our study raises questions about the unique biological or environmental factors that may contribute to widespread genetic homogeneity in *H. kamtschatkana* .

Our finding of panmixia in *H. kamtschatkana* implies that their larvae have strong dispersal ability (Selkoe and Toonen 2011). While larval duration of abalone varies depending on the species and environmental conditions, most species appear to be competent to settle for at least 1–3 weeks (Miyake et al. 2017). In fact, Mccormick et al. (2012) found that closely related *H. rufescens* larvae survived and remained competent for at least 32 days post fertilization; however, post‐settlement survival was optimal only for the first 20 days. Similar results were reported by (Roberts and Lapworth 2001) for *H. iris*. Mccormick et al. (2012) estimated that with a 20‐day competency period and typical water velocities in the California Current of 20 cm s^−1^, *H. rufescens* larvae could be transported up to 350 km, which is well within the range of dispersal estimated by other abalone connectivity studies (Miyake et al. 2017), and indeed, supported by our observation of putative close relatives between the 370 km water distance separating Georgia Strait from West Vancouver Island. Perhaps the most notable potential upper bound to abalone larval dispersal is the ~850 km journey between New Zealand's South Island and the Chatham Islands that must be at least occasionally made by larval *H. iris* to maintain connectivity between populations (Will et al. 2011).

Contemporary gene flow alone may not fully account for the patterns of genetic structure we observed. For example, genetic differentiation in marine broadcast spawners has been hypothesized to arise from semi‐permeable barriers between lineages that evolved partial reproductive isolation during the last glacial maximum (Bierne et al. 2011). Unlike some coastal invertebrates in the region that exhibit genetic differentiation corresponding to biogeographic or hydrodynamic barriers (Kelly and Palumbi 2010), the absence of strong structure in *H. kamtschatkana* could be due to post‐glacial loss of lineages, perhaps leaving behind a single lineage that persisted through post‐glacial recolonization. Moreover, post‐glacial populations may not be in equilibrium between drift and migration, masking genetic differentiation and creating the appearance of high levels of contemporary larval exchange (Kelly and Palumbi 2010; Slatkin 1993). These possibilities highlight the need to consider the longer‐term evolutionary history of the species.

Aside from the effects of short‐ and long‐term population dynamics, other characteristics of abalone may contribute to the limited population structure we observed. Abalone exhibit relatively high levels of genomic polymorphism, which suggests historically large population sizes and high mutation rates (De Wit and Palumbi 2013). Indeed, *H. kamtschatkana* exhibit high allelic diversity and very large effective population sizes (Dimond, Bouma et al. 2022; Withler et al. 2003). These characteristics, common among marine organisms, reduce the effect of genetic drift and require very low levels of gene flow to maintain panmixia. It therefore cannot necessarily be assumed that a lack of population structure in *H. kamtschatkana* has arisen through high dispersal rates (Waples 1998).

Genetic structure in marine species characterized by high gene flow and large effective population size can be challenging to detect amidst noise from various sources of error (Waples 1998). Our technical replicates suggest that genotyping error was extremely low, providing some degree of confidence in our findings. Moreover, despite very low *F*_ST_ values, our analysis revealed evidence of weak isolation by distance, subtle population structure, and areas of relatively reduced migration. Altogether, results point to the Salish Sea as an area of relatively restricted gene flow. Several other genetic studies of marine organisms along the Pacific Coast of North America have also reported genetic differentiation among Salish Sea populations (Buonaccorsi et al. 2002; Dimond, Crim et al. 2022; Drinan et al. 2018; Iwamoto et al. 2015; Jackson and O'Malley 2017; Silliman 2019), suggesting a common mechanism restricting gene flow among a broad swath of species with varying life histories. The Salish Sea is a deep, topographically complex inland sea characterized oceanographically by prevailing estuarine circulation conditions. This circulation transports surface waters above ~60 m depth seaward at ~50 cm s^−1^ through Juan de Fuca Strait, the primary outlet to the Pacific Ocean, approximately 90% of the time during summer and 55% of the time during winter (Thomson, Mihály, and Kulikov 2007). This likely poses a significant barrier to larval delivery from the open ocean. In addition to the Salish Sea, migration analysis using *feems* also indicated relatively low migration rates around Revillagigedo Island in Southeastern Alaska. Like the Salish Sea, Revillagigedo Island is within the Inside Passage, which is protected from the open Pacific by numerous islands, bays, channels and, perhaps most importantly, inlets fed by glacial and snow meltwater. Thus, it may experience reduced gene flow for similar reasons as the Salish Sea (e.g., estuarine circulation pushing surface waters seaward).

Despite the potential for reduced gene flow in the inland waters associated with the Salish Sea and the Inside Passage, the relatively low population‐specific *F*_ST_ and high genetic diversity among these populations, plus Southern Oregon, suggests that the north‐central region between Southern Oregon and Revillagigedo Island represents the ancestral range of the species (Kitada, Nakamichi, and Kishino 2021). Higher population‐specific *F*_ST_ and lower heterozygosity among populations at both the southern (California) and northern (Baranof Island) extent of the range imply these areas represent range expansions to the south and north of the putative north‐central ancestral range of *H. kamtschatkana* . However, given the low sample size from Southern Oregon, we place higher confidence in the region between Washington and southern Southeast Alaska, particularly the Salish Sea and Inside Passage. Notably, this area's numerous islands, bays, and inlets contain abundant rocky subtidal habitat suitable for *H. kamtschatkana* and may have helped facilitate its evolution.

Although we did not find compelling evidence for outlier SNPs implicated as adaptive variants, it is certainly possible that adaptive variation or local adaptation do occur in *H. kamtschatkana* . Reduced representation sequencing techniques such as RADseq have been widely used to evaluate both neutral and adaptive genetic variation (Catchen et al. 2017; Lowry et al. 2017). However, despite their evident superiority over older technology, such as microsatellites, for discerning neutral genetic variation and population structure (e.g., Sunde et al. 2020), these techniques may overlook numerous loci involved in local adaptation due to their limited coverage of the genome, particularly in species with short linkage disequilibrium (Lowry et al. 2017). Future studies may be better equipped to detect such variation using molecular techniques that assess a greater proportion of the genome, such as whole genome resequencing.

The connectivity of *H. kamtschatkana* bears importance to the conservation of the species. Although high gene flow suggests the potential for migration to quickly resupply depleted populations, in reality, signatures of high gene flow in marine populations with large effective sizes can be maintained with just a few individuals per generation. This level of migration is sufficient to maintain connectivity on evolutionary timescales but is negligible in terms of its ability to rebuild dwindling populations within a timeframe relevant to humans (Waples 1998). This justifies the continued effort to restore *H. kamtschatkana* populations via outplants of captive‐bred individuals into the wild, particularly in the Salish Sea, where migration rates are low relative to other areas along the species' range. However, to continue this effort in a region where wild adults to serve as broodstock remain rare, broodstock may need to be sourced from outside of the Salish Sea. Our results suggest that, given the lack of evidence for neutral or adaptive variation, *H. kamtschatkana* broodstock (or cryopreserved gametes) could be sourced from anywhere along the range without significantly impacting the evolutionary history of the species.

## Conflicts of Interest

The authors declare no conflicts of interest.

## Data Availability

Data for this study are available at the NCBI Short Read Archive (SRA) under accession PRJNA1163433 (https://www.ncbi.nlm.nih.gov/bioproject/PRJNA1163433) (Dimond 2024).
